# Prevalence of Cancer Predisposition Germline Variants in Male Breast Cancer Patients: Results of the German Consortium for Hereditary Breast and Ovarian Cancer

**DOI:** 10.3390/cancers14133292

**Published:** 2022-07-05

**Authors:** Muriel Rolfes, Julika Borde, Kathrin Möllenhoff, Mohamad Kayali, Corinna Ernst, Andrea Gehrig, Christian Sutter, Juliane Ramser, Dieter Niederacher, Judit Horváth, Norbert Arnold, Alfons Meindl, Bernd Auber, Andreas Rump, Shan Wang-Gohrke, Julia Ritter, Julia Hentschel, Holger Thiele, Janine Altmüller, Peter Nürnberg, Kerstin Rhiem, Christoph Engel, Barbara Wappenschmidt, Rita K. Schmutzler, Eric Hahnen, Jan Hauke

**Affiliations:** 1 Center for Familial Breast and Ovarian Cancer, Center for Integrated Oncology (CIO), University of Cologne, Faculty of Medicine and University Hospital Cologne, 50937 Cologne, Germany; muriel.rolfes@uk-koeln.de (M.R.); julika.borde@uk-koeln.de (J.B.); mohamad.kayali@uk-koeln.de (M.K.); corinna.ernst@uk-koeln.de (C.E.); kerstin.rhiem@uk-koeln.de (K.R.); barbara.wappenschmidt@uk-koeln.de (B.W.); rita.schmutzler@uk-koeln.de (R.K.S.); jan.hauke@uk-koeln.de (J.H.); 2 Mathematisches Institut, Heinrich-Heine-Universität Duesseldorf, 40225 Duesseldorf, Germany; kathrin.moellenhoff@hhu.de; 3 Institute of Human Genetics, University Wuerzburg, 97074 Wuerzburg, Germany; gehrig@biozentrum.uni-wuerzburg.de; 4 Institute of Human Genetics, University Hospital Heidelberg, 69120 Heidelberg, Germany; christian.sutter@med.uni-heidelberg.de; 5 Department of Gynecology and Obstetrics, Technical University Munich, 80333 Munich, Germany; juliane.ramser@mri.tum.de; 6 Department of Gynecology and Obstetrics, University Hospital Duesseldorf, Heinrich-Heine University Duesseldorf, 40225 Duesseldorf, Germany; niederac@med.uni-duesseldorf.de; 7 Institute for Human Genetics, University Hospital Muenster, 48149 Muenster, Germany; judit.horvath@ukmuenster.de; 8 Institute of Clinical Molecular Biology, Department of Gynecology and Obstetrics, University Hospital of Schleswig-Holstein, Campus Kiel, Christian-Albrechts University Kiel, 24105 Kiel, Germany; norbert.arnold@uksh.de; 9 Department of Gynecology and Obstetrics, LMU Munich, University Hospital Munich, 80337 Munich, Germany; alfons.meindl@med.uni-muenchen.de; 10 Department of Human Genetics, Hannover Medical School, 30645 Hannover, Germany; auber.bernd@mh-hannover.de; 11 Institute for Clinical Genetics, Faculty of Medicine Carl Gustav Carus, TU Dresden, 01062 Dresden, Germany; rump.andreas@klinikum-oldenburg.de; 12 Department of Gynecology and Obstetrics, University of Ulm, 89075 Ulm, Germany; shan.wang-gohrke@uniklinik-ulm.de; 13 Institute of Medical and Human Genetics, Charité-Universitätsmedizin Berlin, 13353 Berlin, Germany; julia.ritter@laborberlin.com; 14 Institute of Human Genetics, University of Leipzig Hospitals and Clinics, 04103 Leipzig, Germany; julia.hentschel@medizin.uni-leipzig.de; 15 Cologne Center for Genomics (CCG) and Center for Molecular Medicine Cologne (CMMC), Faculty of Medicine and University Hospital Cologne, University of Cologne, 50931 Cologne, Germany; hthiele@uni-koeln.de (H.T.); janine.altmueller@bih-charite.de (J.A.); nuernberg@uni-koeln.de (P.N.); 16 Core Facility Genomics, Berlin Institute of Health, Charité-Universitätsmedizin Berlin, 10117 Berlin, Germany; 17 Max Delbrück Center for Molecular Medicine in the Helmholtz Association (MDC), 13125 Berlin, Germany; 18 Institute for Medical Informatics, Statistics and Epidemiology, University of Leipzig, 04107 Leipzig, Germany; christoph.engel@imise.uni-leipzig.de

**Keywords:** breast neoplasms, male breast cancer, breast cancer predisposition genes, genetic testing, familial breast cancer

## Abstract

**Simple Summary:**

Male breast cancer (mBC) is a rare disease associated with a high prevalence of pathogenic germline variants (PVs) in the *BRCA2* gene. However, data regarding other breast cancer (BC) predisposition genes are limited or conflicting. We investigated the prevalence of PVs in *BRCA1/2* and 23 other cancer predisposition genes using an overall study sample of 614 patients with mBC. A high proportion of patients with mBC carried pathogenic germline variants in *BRCA2* (23.0%, 142/614) and *BRCA1* (4.6%, 28/614). A *BRCA1/2* PV prevalence of 11.0% was identified in patients with mBC without a family history of breast and/or ovarian cancer. Patients with *BRCA1/2* PVs did not show an earlier disease onset than those without. Case-control analyses revealed significant associations of protein-truncating variants in *BRCA1*, *BRCA2*, *CHEK2*, *PALB2*, and *ATM* with mBC. Our findings support the benefit of multi-gene panel testing in patients with mBC.

**Abstract:**

Male breast cancer (mBC) is associated with a high prevalence of pathogenic variants (PVs) in the *BRCA2* gene; however, data regarding other BC predisposition genes are limited. In this retrospective multicenter study, we investigated the prevalence of PVs in *BRCA1/2* and 23 non-*BRCA1/2* genes using a sample of 614 patients with mBC, recruited through the centers of the German Consortium for Hereditary Breast and Ovarian Cancer. A high proportion of patients with mBC carried PVs in *BRCA2* (23.0%, 142/614) and *BRCA1* (4.6%, 28/614). The prevalence of *BRCA1/2* PVs was 11.0% in patients with mBC without a family history of breast and/or ovarian cancer. Patients with *BRCA1/2* PVs did not show an earlier disease onset than those without. The predominant clinical presentation of tumor phenotypes was estrogen receptor (ER)-positive, progesterone receptor (PR)-positive, and HER2-negative (77.7%); further, 10.2% of the tumors were triple-positive, and 1.2% were triple-negative. No association was found between ER/PR/HER2 status and *BRCA1/2* PV occurrence. Comparing the prevalence of protein-truncating variants (PTVs) between patients with mBC and control data (ExAC, n = 27,173) revealed significant associations of PTVs in both *BRCA1* and *BRCA2* with mBC (*BRCA1*: OR = 17.04, 95% CI = 10.54–26.82, *p* < 10^−5^; *BRCA2*: OR = 77.71, 95% CI = 58.71–102.33, *p* < 10^−5^). A case-control investigation of 23 non-*BRCA1/2* genes in 340 *BRCA1/2*-negative patients and ExAC controls revealed significant associations of PTVs in *CHEK2*, *PALB2*, and *ATM* with mBC (*CHEK2:* OR = 3.78, 95% CI = 1.59–7.71, *p* = 0.002; *PALB2*: OR = 14.77, 95% CI = 5.02–36.02, *p* < 10^−5^; *ATM*: OR = 3.36, 95% CI = 0.89–8.96, *p* = 0.04). Overall, our findings support the benefit of multi-gene panel testing in patients with mBC irrespective of their family history, age at disease onset, and tumor phenotype.

## 1. Introduction

Male breast cancer (mBC) is a rare disease; less than 1% of all patients with breast cancer (BC) are men [1]. Worldwide, the incidence of mBC is less than 1 per 100,000 man-years [2]. In Germany, approximately 750 men were diagnosed with BC in 2020. In contrast, BC among women is by far the most common tumor entity, with approximately 69.000 newly diagnosed patients annually [3]. Due to its comparative rarity, mBC is routinely excluded from clinical trials on BC. Thus, diagnostic recommendations for mBC have been based on clinical research results primarily focusing on women over the decades. In addition to obvious similarities in the disease course, sex-specific differences reveal, in particular, the need for more specific and separate consideration of mBC [4,5,6]. Examining prospective data has revealed clinically relevant differences in carcinogenesis of mBC, especially the divergent prevalence of germline pathogenic variants (PVs) in the major BC susceptibility genes, *BRCA1/2.* PVs in *BRCA2* represent the most frequent causative gene alteration and have been reported in about 10–16% of patients with mBC [4,7,8]. These are associated with an estimated lifetime risk for mBC of 4–12%, compared with 0.1% in the general male population [9,10]. Barnes et al. demonstrated an average lifetime risk for mBC of 12% for *BRCA2* carriers in their polygenic risk score (PRS)-based risk analyses [11]. In contrast to *BRCA2*, PVs in *BRCA1* are underrepresented in male patients compared with those in female patients with BC [12,13]. After initially disproving the involvement of *BRCA1* in mBC carcinogenesis [14], several studies subsequently suggested an association between mBC and PVs in *BRCA1*, although this association is substantially weaker than that with *BRCA2* [15]. Li et al. recently confirmed the association of *BRCA1* PVs and mBC (risk ratio (RR) = 4.30; 95% confidence interval (CI) = 1.09–16.96) [10].

Recently published guidelines [16,17] concerning managing patients with mBC include, in addition to general therapeutic options, the support of genetic counseling and germline genetic testing for cancer predisposition genes regardless of their family cancer history. Nevertheless, there remains a lack of evidence-based, precise breakdown of possible PVs beyond those in *BRCA2*. As widely confirmed in female patient cohorts [18], several predisposition genes involved in DNA repair pathways such as *ATM*, *CHEK2*, and *PALB2* have also been described in mBC cohorts [19,20,21,22,23]. Overall, data regarding these and other suspected mBC predisposition genes remain limited and have revealed controversial results. Pritzlaff et al. [19] investigated the association of 16 BC risk genes in 708 patients with mBC and found that besides *BRCA2*, *PALB2* and *CHEK2* were associated with mBC risk. No significant association with increased mBC risk was found for *BRCA1* and *ATM.* A study by Rizzolo et al. [20] investigating 503 *BRCA1/2*-negative patients with mBC from Italy confirmed the association of PVs in *PALB2* and found no significant association of PVs in *CHEK2* with increased mBC risk. A study including 102 patients with mBC by Fostira et al. [21] confirmed an association with *BRCA2* and identified *ATM* as the second most frequently mutated risk gene.

As the respective contribution to mBC risk remains controversial, further investigations are needed to evaluate the utility and potential incorporation of multi-gene panel testing in the clinical management of patients with mBC. We performed a multicenter study including 614 patients with mBC recruited for genetic testing through the centers of the German Consortium for Hereditary Breast and Ovarian Cancer (GC-HBOC) to determine PV prevalence in known and suspected BC predisposition genes and to more comprehensively define the genetic predisposition to mBC. We pursued a two-stage approach: first, all patients were screened for PVs in *BRCA1/2*; second, *BRCA1/2*-negative patients were further analyzed for PVs in non-*BRCA1/2* cancer predisposition genes.

## 2. Material and Methods

### 2.1. Study Sample

The overall study sample comprised 614 patients with mBC, diagnosed with unilateral or bilateral BC between 1965 and 2018. The average age at first diagnosis (AAD) was 60 years (range 22–91 years). All patients were recruited from the participating centers of GC-HBOC. For 66.1% of the patients with mBC in the overall study sample (406/614), a positive family history (FH) for BC and/or ovarian cancer (OC) was reported. Positive FH was defined as at least one known relative with BC or OC, irrespective of the AAD of the relative(s) with BC or OC. Among the patients with mBC, 32.6% reported no FH of BC/OC (200/614). Data regarding BC/OC FH were missing for 8/614 of the patients with mBC (1.3%). *BRCA1/2* germline analysis was performed in a routine diagnostic setting between 1995 and 2019. All patients were tested for germline mutations after mBC was diagnosed. A high proportion (27.7%) of patients with mBC (170/614) carried PVs in *BRCA1/2*. Of the 614 patients, 586 were considered index patients, with no prior testing of another family member. The remaining 28 mBC patients, all with a positive FH, were analyzed for a known pathogenic *BRCA1/2* family mutation only. Of those, 22 were tested positive. Of the 444 patients with mBC without PVs in *BRCA1/2*, 104 were excluded from further analyses because of missing DNA samples or lack of patient consent. The remaining 340 patients with mBC who had previously tested negative for PVs in *BRCA1/2* were screened for PVs in 23 established or suspected non-*BRCA1/2* BC predisposition genes. Genotype and phenotype data were retrieved from the centralized GC-HBOC patient database (BRCA2006/HerediCaRe, accessed on 20 May 2019).

### 2.2. Gene Selection and Next-Generation-Sequencing (NGS) Analysis

Genomic DNA was isolated from venous blood. Regarding non-*BRCA1/2* predisposition gene analyses, approximately one-third (102/340) of the patient genetic data originated from a comprehensive analysis of gene panel testing previously performed at the GC-HBOC centers in a routine diagnostic setting using the customized hybridization capture-based TruRisk^®^ gene panel of the GC-HBOC for target enrichment. Another customized multi-gene panel was used for the remaining 238 patients (Agilent SureSelect^XT^, Santa Clara, CA, USA). Both multi-gene panels covered the entire coding regions of 23 established or suspected non-*BRCA1/2* BC predisposition genes (*ATM*, NM_000051.3; *BARD1*, NM_000465.3; *BRIP1*, NM_032043.2; *CDH1*, NM_004360.4; *CHEK2*, NM_007194.3; *FAM175A*, NM_139076.2; *FANCM*, NM_020937.3; *MLH1*, NM_000249.3; *MRE11A*, NM_005591.3; *MSH2*, NM_000251.2; *MSH6*, NM_000179.2; *MUTYH*, NM_0011428425.1; *NBN*, NM_002485.4; *PALB2*, NM_024675.3; *PMS2*, NM_000535.5; *PTEN*, NM_000314.4; *RAD50*, NM_005732.3; *RAD51C*, NM_058216.2; *RAD51D*, NM_002878.3; *RINT1*, NM_021930.4; *STK11*, NM_000455.4; *TP53*, NM_000546.5; *XRCC2*, NM_005431.1). Quantified libraries were sequenced on Illumina NGS devices (HiSeq 4000 or NovaSeq 6000, San Diego, CA, USA) at the Cologne Center for Genomics (CCG). Bioinformatic analyses were performed using the Varbank v.2.26 (Cologne Center for Genomics, Cologne, Germany) pipeline of the CCG.

### 2.3. Variant Annotation and Classification

The sequencing reads were mapped against human reference genome GRCh38. Mean coverage of at least 100× was chosen as the sequencing quality filter threshold. The Alamut Visual version 2.13 analysis software tool (Interactive Biosoftware, Rouen, France) was used for variant annotation and integration of current ClinVar classifications. Variant classification was performed using the GC-HBOC criteria for the classification of germline sequence variants in risk genes for hereditary BC and OC [24], based on the interpretive guidelines published by the Evidence-Based Network for the Interpretation of Germline Mutant Alleles (ENIGMA) and the American College of Medical Genetics and Genomics (ACMG) [25]. As proposed by the International Agency for Research on Cancer (IARC) [26], a five-tier classification system was applied. This grading system defines variants as pathogenic (class 5), likely pathogenic (class 4), variant of uncertain significance (VUS, class 3), likely benign (class 2), or benign (class 1). Protein-truncating germline variants (PTVs) were defined as nonsense, frameshift, or essential splice site variants affecting the invariant splice sites or the last nucleotide of an exon. PVs (pathogenic variants) included PTVs, (likely) pathogenic missense variants, and (likely) pathogenic copy number variations (CNVs). ExomeDepth v1.1.15 was used for CNV prediction [27] and predicted CNVs were verified by multiplex ligation-dependent probe amplification (MLPA) using specific P041 (*ATM*), P042 (*ATM*), and P190 (*CHEK2*) SALSA^®^ MLPA^®^ kits (MRC Holland, Amsterdam, The Netherlands). All remaining PVs were confirmed by Sanger sequencing.

### 2.4. Control Sample and Statistical Analysis

To investigate the association of mBC with PTVs in suspected cancer predisposition genes, a case-control analysis was conducted using univariate logistic regression analysis to estimate the odds ratios (ORs) and corresponding 95% confidence intervals (CIs). Publicly available genomic variant data from 27,173 individuals of non-Finnish European ancestry from the Exome Aggregation Consortium (ExAC) [28], excluding samples from The Cancer Genome Atlas (TCGA), were included as control data. Finnish individuals were excluded due to a high prevalence of founder mutations in the Finnish population [29], which may cause a bias in the case-control analysis. Statistical analyses were performed using R v3.6 (R Core Team (2021)). R: A language and environment for statistical computing. R Foundation for Statistical Computing, Vienna, Austria. URL: https://www.R-project.org/ (accessed on 23 February 2022). All statistical tests were two-sided, with *p*-values < 0.05 considered statistically significant.

## 3. Results

### 3.1. Prevalence of BRCA1/2 Pathogenic Variants and Cancer Characteristics in the Overall Study Sample

From the overall study sample of 614 patients with mBC, 170 (27.7%) patients carried a PV in *BRCA1* or *BRCA2*, with a notably higher proportion of *BRCA2* PV carriers (142/614, 23.1%) than *BRCA1* PV carriers (28/614, 4.6%). In *BRCA2*, the c.1813dup PV was most frequent (12/142). In *BRCA1*, the c.5266dup variant accounted for one-quarter of all *BRCA1* PVs (7/28). The mean AAD of *BRCA1* PV carriers, as well as that of *BRCA2* PV carriers, was 62 years (*BRCA1*: range 33–82 years; *BRCA2:* range 37–83 years), with a statistically significant difference observed compared with *BRCA1/2*-negative patients (mean 59 years, range 22–91 years, Welch’s *t*-test *p* = 0.005). Among 606 patients with mBC whose BC/OC FH status was known, the prevalence of *BRCA1/2* PVs was significantly higher in patients with BC/OC FH than in those without (154/460 (33.5%) vs. 16/146 (11.0%), Fisher’s exact test *p* < 10^−7^). A statistically significant association between BC/OC FH and the occurrence of PVs was found the *BRCA2* gene (128/460 (27.8%) vs. 14/146 (9.6%), OR = 3.63, 95% CI = 1.99–7.08, Fisher’s exact test *p* < 10^−5^) and for the *BRCA1* gene (26/460 (5.6%) vs. 2/146 (1.4%), *p* = 0.04). A high proportion of PVs in the *BRCA2* gene was observed in bilaterally affected patients with mBC, though with no statistical significance compared with the prevalence in unilaterally affected patients with mBC (*p* = 0.112); of the 28 bilaterally affected patients with mBC (Table 1), 10 patients (35.7%) carried PVs in *BRCA2* compared with 132/586 (22.5%) in unilaterally affected patients.

Information on tumor estrogen receptor (ER) and progesterone receptor (PR) status was available for 407 patients with mBC (66%) among the overall study sample of 614 patients with mBC. The predominant clinical presentation of tumor phenotypes was ER-positive and PR-positive (359/407, 88.2%); further, 35/407 (8.6%) were ER-positive and PR-negative. Only 13/407 (3.2%) tumors were ER-negative, of which seven were PR-positive and six were PR-negative. Among the 407 patients with mBC with known ER and PR status, human epidermal growth factor receptor 2 (HER2) status was documented in 323 patients. Most patients were ER-positive, PR-positive, and HER2-negative (251/323, 77.7%); further, 33/323 (10.2%) were triple-positive, and 4/323 (1.2%) were triple-negative (Figure S1). Neither AAD nor *BRCA1/2* PV carrier status was associated with ER, PR, or HER2 status (Figure S2, and Table S1).

### 3.2. Prevalence of Pathogenic Variants in BRCA1/2-Negative Patients with mBC

**Figure 1:**
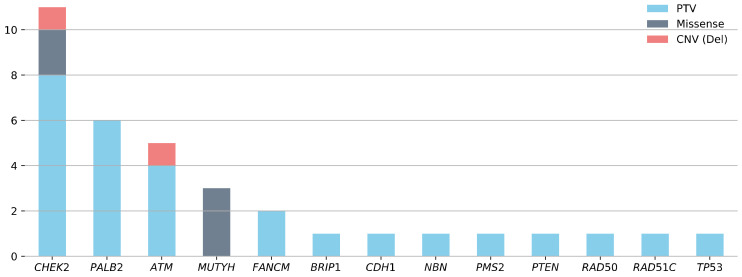
Gene-specific prevalence of heterozygous pathogenic germline variants (PVs) in 340 patients with *BRCA1/2*-negative male breast cancer.

In the overall study sample, 72.3% (444/614) of patients tested negative for PVs in *BRCA1/2.* Of these patients, 340 were screened for PVs among 23 additional non-*BRCA1/2* cancer predisposition genes. This subgroup, with a mean AAD of 60 years (range 27–91), comprised 328 unilaterally and 12 bilaterally affected patients with mBC (Table 1). A BC/OC FH was reported in 69.7% (235/337) of these patients (Table 1, no BC/OC FH data were available for three patients). In the *BRCA1/2*-negative study sample, 9.4% (32/340) of the patients with mBC, all unilaterally affected, carried at least one PV in the 23 (suspected) non-*BRCA1/2* cancer predisposition genes (Figure 1).

Overall, 35 PVs were identified in the 13 genes (Figure 1). Three patients with mBC were double PV carriers (*CHEK2*/*ATM*, *NBN*/*RAD50*, *PALB2*/*TP53*). PVs in *CHEK2* were the most frequent and were identified in 3.2% (11/340) of the *BRCA1/2*-negative patients with mBC. The most prevalent PV in the *CHEK2* gene was the c.1100delC variant observed in six of 11 patients. The second highest PV prevalence was in *PALB2* (1.8%, 6/340), followed by *ATM* (1.5%, 5/340). The prevalence of PVs in other investigated genes was less than 1% each (*MUTYH* (3/340), *FANCM* (2/340), *BRIP1* (1/340), *CDH1* (1/340)*, NBN* (1/340), *PMS2* (1/340), *PTEN* (1/340), *RAD50* (1/340), *RAD51C* (1/340), *TP53* (1/340)). No PVs were identified in *BARD1, FAM175A, MLH1, MRE11A, MSH2, MSH6, RAD51D, RINT1, STK11*, or *XRCC2* (Table S1).

### 3.3. Associations between mBC and Protein-Truncating Variants in BRCA1/2 and Non-BRCA1/2 Cancer Predisposition Genes

**Figure 2:**
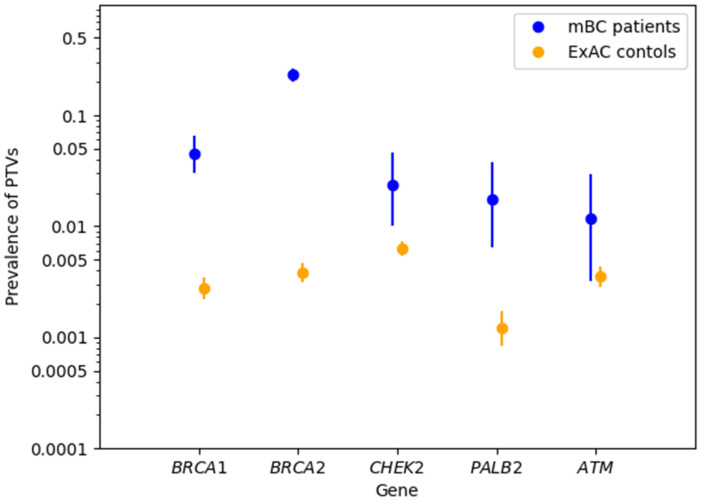
Prevalence of protein-truncating variants (PTVs) with binomial 95% confidence intervals (CIs) per gene in patients with male breast cancer and in ExAC controls.

Based on the sequencing results from the publicly available ExAC control dataset [28], case-control analyses were performed to assess associations between mBC and PTVs in *BRCA1/2* and selected non-*BRCA1/2* genes. PTVs in both *BRCA2* and *BRCA1* were significantly associated with mBC (*BRCA2*: OR = 77.41, 95% CI = 58.71–102.33, Fisher’s exact test *p* < 0.0001; *BRCA1*: OR = 17.04, 95% CI = 10.54–26.82, *p* < 0.0001) (Table 2). Among the 340 patients with *BRCA1/2*-negative mBC, 25 (7.35%) carried PTVs in 10 non-*BRCA1/2* genes (Table S1). At a gene-specific level, the prevalence of PTVs in *PALB2* was significantly higher in patients with mBC than in the ExAC controls (6/340 (1.76%) vs. 33/27,173 (0.12%); OR = 14.77; 95% CI = 5.02–36.02; *p* < 0.0001, Table 2). Statistically significant associations were also observed between mBC and PTVs in *CHEK2* (OR = 3.78; 95% CI = 1.59–7.71; *p* = 0.002) and *ATM* (OR = 3.36; 95% CI = 0.89–8.96; *p* = 0.04) (Table 2). Notably, the number of identified PTVs was low (≤8) for each of the non-*BRCA1/2* genes, and the 95% CIs of the observed PTV prevalence did not reach a distinction for the *ATM* gene, which only affected four patients with mBC (Figure 2). Beyond *PALB2, CHEK2*, and *ATM*, the prevalence of PTVs in the other examined genes was too low for meaningful statistical analysis.

### 3.4. Pathogenic Variants in Cancer Predisposition Genes according to Cancer Family History, Age at Diagnosis, and Tumor Characteristics

**Figure 3:**
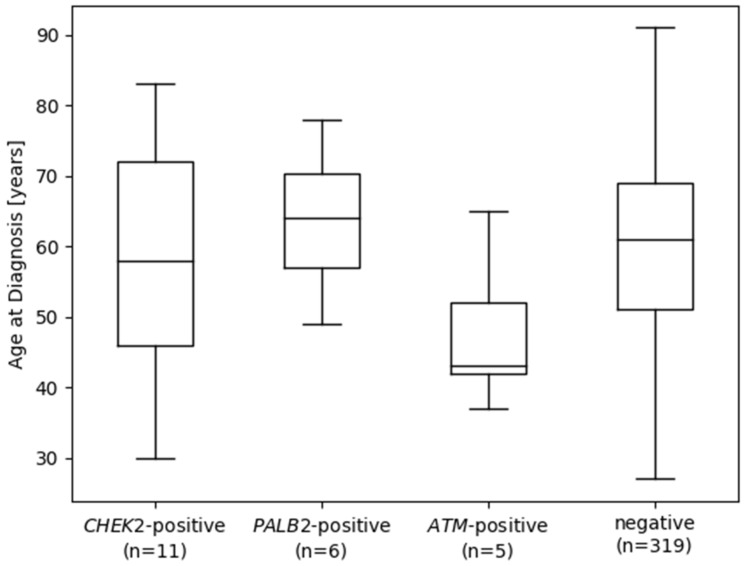
Age at diagnosis (AAD) of male breast cancer in *BRCA1/2*-negative individuals based on germline pathogenic variant (PV) status in the *CHEK2*, PALB2, and *ATM* genes. One individual (AAD = 37 years) carried PVs in both *CHEK2* and *ATM*. The term “negative” indicates individuals without PVs in *BRCA1/2*, *CHEK2*, *PALB2*, and *ATM*.

In the subset of 340 *BRCA1/2*-negative patients with mBC, the overall prevalence of PVs in patients with a BC/OC FH was 9.8% (23/235), and that in patients without a BC/OC FH was 8.8% (9/102) (23/235 vs. 9/102; Fisher’s exact test *p* = 0.84). When focusing on *ATM*, *CHEK2*, and *PALB2*, the prevalence of PVs in patients with BC/OC FH was 7.7% (18/235), whereas that in patients without BC/OC FH was 2.9% (3/102) (Fisher’s exact test, *p* = 0.14). The mean AAD of patients with mBC was considerably lower in carriers of a PV in *ATM* (Figure 3) compared with that in patients without (mean AAD *ATM*-positive 47.8 years (n = 5) vs. *ATM*-negative 59.9 years (n = 335), Welch’s *t*-test *p* = 0.07). Linear regression analysis with the AAD of mBC in years as the outcome revealed a statistically significant association of PVs in the *ATM* gene with younger AAD (Table 3) under adjustment for the presence of BC/OC FH, whereas PVs in *CHEK2* and *PALB2* showed no statistically significant association with AAD.

## 4. Discussion

Comprehensive germline genetic testing is a prerequisite in targeted risk-adjusted surveillance programs for early cancer detection and targeted therapies in the context of precision oncology. Therefore, germline genetic testing represents a globally established standard; however, it primarily remains limited to women with BC. Research on PARP inhibitors beyond *BRCA1/2*-mutated carcinomas, for example, is pioneering the field of personalized medicine [30]. Thus, sex-specific consideration of distinct tumor entities in conjunction with their respective predominant germline defects is particularly important. In our multicenter study, we investigated genetic susceptibility to mBC by evaluating the prevalence of PVs in *BRCA1/2* using a study sample of 614 patients with mBC and screening their blood-derived DNA samples with multi-gene panel analyses for potentially relevant PVs in 23 non-*BRCA1/2* genes. As the largest nationwide sample, with 340 of 614 patients with mBC undergoing comprehensive genetic screening, this study contributes to decoding the genetic predisposition to mBC and reassessing the previously obtained contradictory study results. As expected, the role of *BRCA2* as a key risk gene for mBC was highlighted. [12] In the overall study sample, 23.1% (142/614) of patients with mBC carried a PV in *BRCA2*. Further, as PVs in *BRCA1* accounted for 4.6% of the cases, *BRCA1* was the second most frequently altered BC predisposition gene. While *BRCA2* represents an established BC risk gene for both female and male BC, literature regarding the association between *BRCA1* and mBC is limited. The results of our study are in line with those of Li et al. [10], who were among the first to demonstrate a significant association between mBC and PVs in *BRCA1* (OR = 17.04, 95% CI 10.54–26.82, *p* < 10^−5^).

Consistent with the comprehensive population-specific analyses conducted by Rebbeck et al., we demonstrated that the PVs, *BRCA2* c.1813dup and *BRCA1* c.5266dup, are the most common variants in the mutational spectrum of mBC. [31] The investigation of available information on ER, PR, and HER2 status revealed predominantly ER-positive, PR-positive, and HER2-negative tumor presentation in the studied sample, consistent with previous findings characterizing the general cancer type in virile breast carcinoma. [32] In this context, Silvestri et al. previously showed that *BRCA1/2*-positive mBCs were more likely to be ER-positive, PR-positive, and non-triple negative, compared with BC in female *BRCA1/2* carriers, suggesting that susceptibility to hereditary BC may be influenced by hormonal background differences between male and female *BRCA1/2* mutation carriers [12]. In our study focusing on mBC only, no association of the ER/PR/HER2 status with *BRCA1/2* PV occurrence was observed.

Among the non-*BRCA1/2* genes, our study confirmed a significantly increased risk association between mBC and PTVs in the BC predisposition genes *PALB2, CHEK2*, and *ATM.* This finding may be considered in mBC risk prediction models such as BOADICEA (Breast and Ovarian Analysis of Disease Incidence and Carrier Estimation Algorithm) [33]. We demonstrated the strongest association of mBC in carriers of PVs in *PALB2* with an OR of 14.77 (*p* < 0.0001) compared with that in ExAC controls. As a functional partner and localizer of *BRCA2*, the tumor suppressor gene *PALB2* is critically involved in the homologous recombination (HR) repair mechanism of double-strand breaks [34]. Consistent with previously reported associations between PVs in *PALB2* and mBC (with corresponding ORs ranging from 9.63–17.30) [20], our findings contribute to the accumulating evidence for the relevance of *PALB2* in genetic testing for hereditary mBC [35,36]. The role of *CHEK2* in mBC has been discussed contradictorily in recent studies. A study by Pritzlaff et al. defined *CHEK2* as a moderate-risk gene [19], whereas other analyses could not confirm an association of *CHEK2* with genetic mBC predisposition [20,21]. In the present investigation, *CHEK2* represented the gene most frequently affected by PVs apart from *BRCA1/2*, with a PV prevalence of 3.2%, and a statistically significant association between PTVs in *CHEK2* and mBC risk was confirmed (OR = 3.78, 95% CI = 1.59–7.71, *p* = 0.002). Furthermore, a statistically significant association was found between PTVs in *ATM* and mBC (mBC vs. ExAC controls OR = 3.36, 95% CI: 0.89–8.96, *p* = 0.04), and the data indicate a younger AAD in *ATM* PV carriers. Future studies with larger sample sizes are required to conclusively assess the role of *ATM* in mBC development, as our study cohort included only 5 *ATM* PV carriers. In contrast to the strikingly divergent frequencies of PTVs in *BRCA1/2* in male and female patients with BC, the prevalence of PTVs in other predisposition genes, including *PALB2*, *CHEK2*, and *ATM*, were comparable in both genders [18,37]. Patients with *BRCA1/2* PVs did not show an earlier disease onset than those without. Except for patients with mBC carrying pathogenic *ATM* variants, who showed a younger AAD of BC in our study sample, AAD does not seem to be a useful indicator of genetic mBC predisposition. Further, we could not support the results of Pritzlaff et al., who suggested that *CHEK2* c.1100delC carriers had a significantly younger AAD compared with that men in carrying PVs in other risk genes [19]. Our results support the NCCN guidelines for male invasive BC version 5.2020, which recommend genetic testing for all men with BC, regardless of their BC/OC FH. Based on this recommendation, we propose to expand the inclusion criteria for testing for genetic to all patients with mBC in Germany, regardless of their AAD, FH, or tumor phenotype. In conclusion, our study results continue to pursue the overall objective of better understanding genetic predisposition to mBC for developing appropriate clinical approaches for sex-specific risk prediction, which could lead to more targeted screening and treatment programs for male PV carriers. It remains to be determined whether the germline mutation status in *BRCA1/2*, *ATM*, *CHEK2* and *PALB2* predicts favourable or unfavourable targeted therapy response in mBC patients, e.g., regarding PARP or CDK4/6 inhibitors [38].

This study has limitations. Our study sample largely comprised patients with mBC who met the GC-HBOC inclusion criteria for germline testing. Therefore, these study results should be validated in a larger study sample of unselected patients with mBC. Further, the prevalence of PTVs in unaffected individuals was retrieved from ExAC non-Finnish Europeans (NFE) under the exclusion of TCGA instead of matched controls, which may have caused bias. The mBC patients who were initially tested for *BRCA1/2* mutations only and tested positive were not analyzed further using a more comprehensive gene panel.

## 5. Conclusions

In addition to PVs in the established mBC risk gene *BRCA2*, PVs in *BRCA1* are particularly associated with an increased risk of mBC. Our results further suggest the role of *PALB2*, *CHEK2*, and *ATM* in mBC predisposition, and support the benefit of multi-gene panel testing in patients with mBC. Due to the high prevalence of *BRCA1/2* PVs even in the absence of a BC/OC FH, our data provide a rationale to offer genetic counseling and multi-gene panel testing to all patients with mBC.

## Figures and Tables

**Table 1:** Patient and cancer characteristics in the overall study sample (n = 614) stratified by pathogenic variant (PV) status, family history (FH), age at first diagnosis (AAD). BC = breast cancer, ER = estrogen receptor, mBC = male breast cancer, PR = progesterone receptor.

Subgroup	Overall Study Sample (%)	*BRCA1/2-* Positive	*BRCA1-* Positive	*BRCA2-* Positive	*BRCA1/2* Negative Patients Further Investigated	Carriers of Pathogenic Variants in 23 Non-*BRCA1/2*-Genes (%)
patients with mBC	614 (100)	170	28	142	340	32 (9.4)
unilateral BC	586 (95.4)	160	28	132	328	32 (9.8)
bilateral BC	28 (4.6)	10	0	10	12	0 (0)
BC/OC FH *	460 (75.9)	154	26	128	235	23 (9.8)
no BC/OC FH *	146 (24.1)	16	2	14	102	9 (8.8)
mean AAD (range) *	60 (22–91)	62 (33–83)	62 (33–82)	62 (37–83)	60 (27–91)	58 (30–83)
AAD < 40 years	30 (4.9)	4 (2.4)	1	3	18	2 (11.2)
AAD 40–49 years	90 (14.8)	18 (10.6)	4	14	55	8 (14.5)
AAD 50–59 years	163 (26.8)	42 (24.7)	5	37	89	7 (7.9)
AAD 60–69 years	183 (30.0)	61 (35.9)	9	52	97	7 (7.2)
AAD 70–79 years	125 (20.5)	40 (23.5)	8	32	69	7 (10.1)
AAD > 80 years	18 (3.0)	5 [2.9]	1	4	12	1 (8.3)
ER/PR-status available (%)	407 (100)	107 (100)	17 (100)	90 (100)	243 (100)	24 (100)
HER2-status available (%)	323 (100)	90 (100)	15 (100)	75 (100)	200 (100)	21 (100)
ER-positive (%)	394 (96.8)	105 (98.1)	17 (100)	88 (97.8)	234 (96.3)	24 (100)
ER-negative (%)	13 (3.2)	2 (1.9)	0 (0)	2 (2.2)	9 (3.7)	0 (0)
PR-positive (%)	366 (89.9)	94 (87.9)	14 (82.4)	80 (88.9)	219 (90.1)	23 (96.0)
PR-negative (%)	41 (10.1)	13 (12.1)	3 (17.6)	10 (11.1)	24 (9.9)	1 (4.2)
HER2-positive (%)	38 (11.7)	13 (14.4)	2 (13.3)	11 (14.7)	20 (10.0)	4 (19.0)
HER2-negative (%)	285 (88.2)	77 (85.6)	13 (86.7)	64 (85.3)	180 (90.0)	17 (81.0)

* total missing information regarding AAD (n = 5) and missing information regarding FH (n = 8).

**Table 2:** Prevalence of protein-truncating variants (PTVs) in (suspected) cancer predisposition genes in patients with mBC compared with the control dataset (ExAC). Mutation carrier frequencies are shown in parentheses. Univariate logistic regression analysis was performed to estimate the odds ratios (OR) and corresponding 95% confidence intervals (CI).

Gene	mBC PTVs (%)	Patients with mBC	ExAC Controls n = 27,173 (%)	mBC vs. ExAC	
				OR (95% CI)	*p* *
*BRCA2*	142 (23.13)	614	105 (0.39)	77.41 (58.71–102.33)	<10^−5^
*BRCA1*	28 (4.56)	614	76 (0.28)	17.04 (10.54–26.82)	<10^−5^
*CHEK2*	8 (2.35)	340	172 (0.63)	3.78 (1.59–7.71)	0.002
*PALB2*	6 (1.76)	340	33 (0.12)	14.77 (5.02–36.02)	<10^−5^
*ATM*	4 (1.18)	340	96 (0.35)	3.36 (0.89–8.96)	0.04
*FANCM*	2 (0.59)	340	184 (0.68)	-	-
*BRIP1*	1 (0.29)	340	59 (0.22)	-	-
*CDH1*	1 (0.29)	340	2 (0.00)	-	-
*NBN* **	1 (0.29)	340	42 (0.15)	-	-
*PTEN*	1 (0.29)	340	1 (0.00)	-	-
*RAD50* **	1 (0.29)	340	84 (0.31)	-	-
*RAD51C*	1 (0.29)	340	34 (0.13)	-	-

* Fisher’s exact test; ** one patient carried two PTVs (NBN/RAD50).

**Table 3:** Linear regression analysis results with age at first diagnosis (AAD) of male breast cancer (mBC) with years as the outcome in 337 *BRCA1/2*-negative patients with mBC. Gene-wise covariates refer to pathogenic variant status (1: pathogenic variant, 0: no pathogenic variant). FH = Breast/ovarian cancer family history (1: yes 0: no).

Covariat	β	95% CI	*p*
FH	1.78	−0.97–4.52	0.20
*ATM*	−11.87	−22.35–−1.39	0.03
*CHEK2*	−1.96	−9.09–5.17	0.59
*PALB2*	3.55	−5.98–13.07	0.46

## Data Availability

The data presented in this study are available on request from the corresponding author. The data are not publicly available due to privacy.

## Supplementary Materials

The following supporting information can be downloaded at: https://www.mdpi.com/article/10.3390/cancers14133292/s1, Figure S1: Venn diagram of hormone receptor status of 323 indivduals with male breast cancer. ER+/−: Estrogen receptor positive/negative. PR+/−: Progesterone receptor positive/negative. HER2 +/−: human epidermal growth factor receptor 2 positive/negative; Figure S2: Proportion of hormone receptor-positve tumors per age at first diagnosis (AAD) in 323 mBC patients. ER+: Estrogen receptor positive. PR+: Progesterone receptor positive. HER2 +: human epidermal growth factor receptor 2 positive; Table S1: Pathogenic variants (PVs) in non-*BRCA1/2* genes detected in 32/340 mBC patients. AAD = age at first diagnosis, CNV = copy number variation, PTV = protein truncating variant, mBC = male breast cancer; Table S2: Binary logistic regression analyses with tumor receptor status as the outcome (1 = positive; 0 = negative) for 323 individuals with male breast cancer. Gene-wise covariates refer to pathogenic variant carrier status (1 = carrier; 0 = non-carrier). CI = confidence interval. SE = standard error.

## Abbreviations

AAD	age at first diagnosis
ACMG	American College of Medical Genetics
BC	breast cancer
CCG	Cologne Center for Genomics
CI	confidence interval
CNV	copy number variation
ER	estrogen receptor
ExAC	Exome Aggregation Consortium
FH	family history
GC-HBOC	German Consortium for Hereditary Breast and Ovarian Cancer
HER2	human epidermal growth factor receptor 2
IARC	International Agency for Research on Cancer
mBC	male breast cancer
MLPA	multiplex ligation-dependent probe amplification
NCCN	National Comprehensive Cancer Network
NFE	non-Finnish European
NGS	next-generation sequencing
PR	progesterone receptor
PTV	protein-truncating variant
PV	pathogenic variant
OC	ovarian cancer
OR	odds ratio
TCGA	The Cancer Genome Atlas
VUS	variant of uncertain significance
