# Supplementary material for: Prevalence of Cancer Predisposition Germline Variants in Male Breast Cancer Patients: Results of the German Consortium for Hereditary Breast and Ovarian Cancer
Source: Cancers (Basel). 2022 Jul 5;14(13):3292. doi: 10.3390/cancers14133292 (PMC9265404; doi:10.3390/cancers14133292)
Supplement: Supplementary file 1 [file cancers-14-03292-s001.zip › cancers-1740746-supplementary.pdf]

cancers MDPI

Supplementary

Prevalence of cancer predisposition germline variants in male breast cancer patients: Results of the German Consortium for Hereditary Breast and Ovarian Cancer

Table S1. Pathogenic variants (PVs) in non-BRCA1/2 genes detected in 32/340 mBC patients. AAD = age at first diagnosis, CNV = copy number variation, PTV = protein truncating variant, mBC = male breast cancer.

Gene	exon/ intron location	c.DNA position	HGVS (protein)	mutation class	mutation type	AAD	BC/OC family history
ATM	exon 40	c.5932G>T	p.Glu1978*	class 5	PTV	52	Yes
ATM	exon 50	c.7327C>T	p.Arg2443*	class 5	PTV	65	Yes
ATM	exon 60	c.8690del	p.Gly2897Alafs*41	class 5	PTV	42	Yes
ATM	exon 61	c.8793T>A	p.Cys2931*	class 5	PTV	43	Yes
ATM[1]	/	whole gene deletion	p.?	class 5	CNV	37	No
BRIP1	exon 16	c.2273dup	p.Ala759Serfs*6	class 5	PTV	57	Yes
CDH1	exon 8	c.1131delC	p.Thr378Profs*15	class 5	PTV	70	Yes
CHEK2	exon 3	c.349A>G	p.Arg117Gly	class 4	MISSENSE	49	Yes
CHEK2	intron 6	c.792+2T>C	p.?	class 4	PTV	30	No
CHEK2	exon 9/10	deletion exon 9/10	p.?		CNV	72	No
CHEK2	exon 11	c.1100delC	p.Thr367Metfs*15	class 5	PTV	72	Yes
CHEK2	exon 11	c.1100delC	p.Thr367Metfs*15	class 5	PTV	47	Yes
CHEK2	exon 11	c.1100delC	p.Thr367Metfs*15	class 5	PTV	45	Yes
CHEK2	exon 11	c.1100delC	p.Thr367Metfs*15	class 5	PTV	73	Yes
CHEK2	exon 11	c.1100delC	p.Thr367Metfs*15	class 5	PTV	58	Yes
CHEK2	exon 11	c.1100delC	p.Thr367Metfs*15	class 5	PTV	83	No
CHEK2	exon 11	c.1169A>C	p.Tyr390Ser	class 4	MISSENSE	58	Yes
CHEK2[1]	exon 11	c.1238T>G	p.Leu413*	class 5	PTV	37	No
FANCM	exon 14	c.3979_3980delCA	p.Gln1327Valfs*16	class 5	PTV	49	No
FANCM	exon 20	c.5101C>T	p.Gln1701*	class 5	PTV	73	No
MUTYH	exon 7	c.536A>G	p.Tyr179Cys	class 5	MISSENSE	63	No
MUTYH	exon 13	c.1187G>A	p.Gly396Asp	class 4	MISSENSE	60	Yes
MUTYH	exon 13	c.1187G>A	p.Gly396Asp	class 4	MISSENSE	64	Yes
NBN[2]	intron 8	c.995-2A>G		class 4	PTV	40	No
PALB2	exon 2	c.50T>G	p.Leu17*	class 5	PTV	71	Yes
PALB2	exon 3	c.172_175delTTGT	p.Gln60Argfs*7	class 5	PTV	56	No
PALB2	exon 3	c.172_175delTTGT	p.Gln60Argfs*7	class 5	PTV	49	Yes
PALB2	exon 4	c.509_510delGA	p.Arg170Ilefs*14	class 5	PTV	60	Yes
PALB2[3]	exon 4	c.886dup	p.Met296Asnfs*7	class 5	PTV	68	Yes
PALB2	exon 4	c.1597_1603dup	p.Ser535Asnfs*3	class 5	PTV	78	Yes
PMS2	exon 13	c.2249G>A	p.Gly750Asp	class 4	MISSENSE	66	Yes
PTEN	exon 7	c.697C>T	p.Arg233*	class 4	PTV	57	No
RAD50[2]	exon 4	c.541dup	p.Ser181Phefs*7	class 4	PTV	40	No
RAD51C	intron 6	c.905-2_905-1delAG	p.?	class 4	PTV	51	Yes
TP53[3]	exon 8	c.836G>A	p.Gly279Glu	class 4	MISSENSE	68	Yes

[1] Double mutation carrier: *CHEK2*/*ATM* deletion (CNV)

[2] Double mutation carrier: *NBN*/ *RAD50*.

[3] Double mutation carrier: *PALB2*/*TP53*.

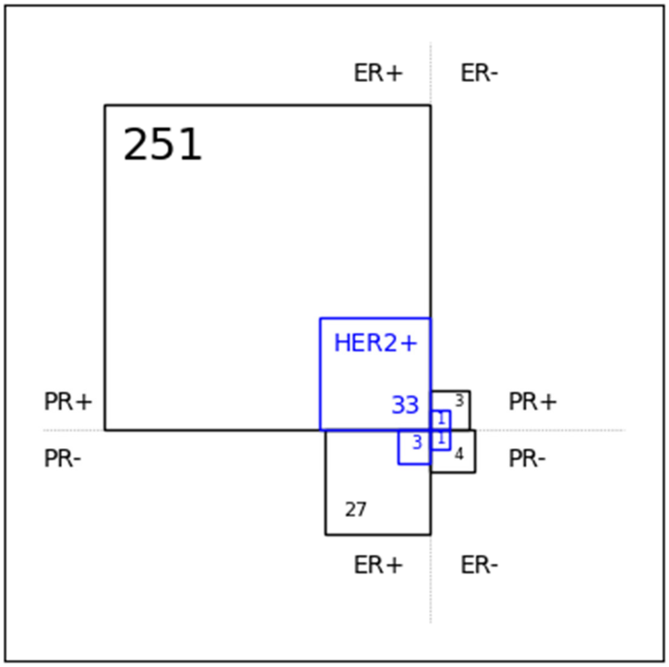

Figure S1. Venn diagram of hormone receptor status of 323 indivduals with male breast cancer. ER+/−: Estrogen receptor positive/negative. PR+/−: Progesterone receptor positive/negative. HER2 +/−: human epidermal growth factor receptor 2 positive/negative.

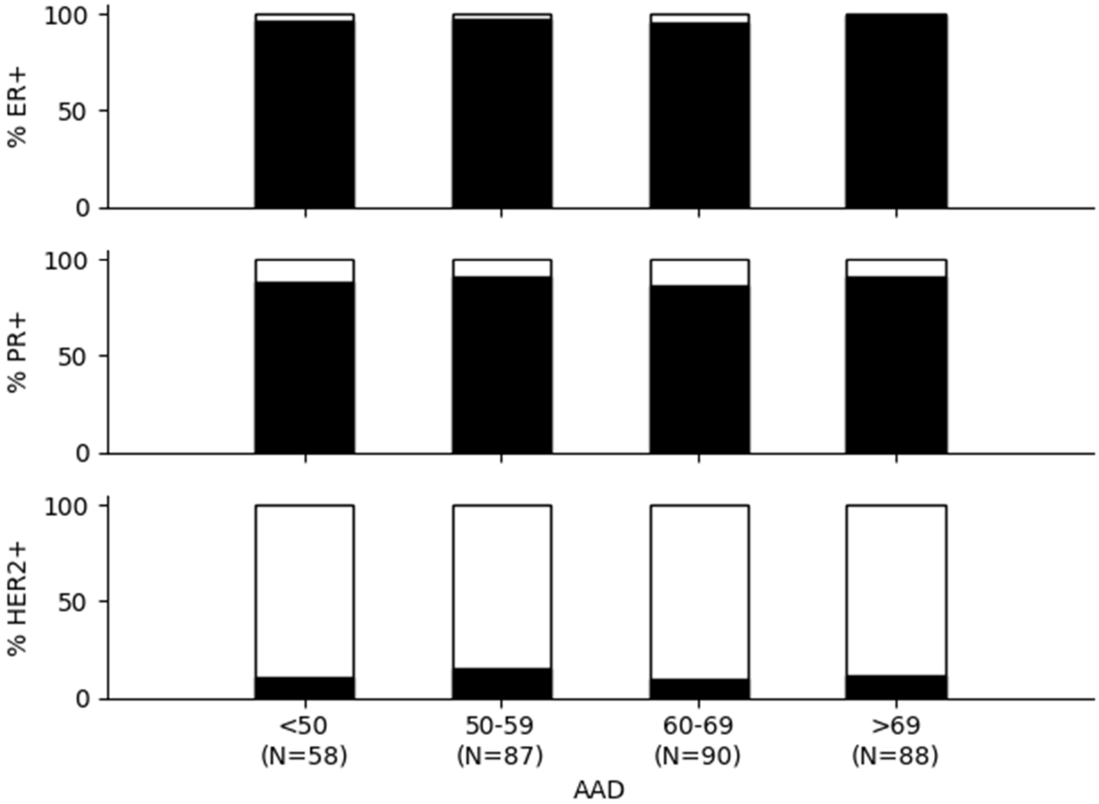

Figure S2. Proportion of hormone receptor-positve tumors per age at first diagnosis (AAD) in 323 mBC patients. ER+: Estrogen receptor positive. PR+: Progesterone receptor positive. HER2 +: human epidermal growth factor receptor 2 positive.

Table S2. Binary logistic regression analyses with tumor receptor status as the outcome (1 := positive; 0 := negative) for 323 individuals with male breast cancer. Gene-wise covariates refer to pathogenic variant carrier status (1 := carrier; 0 := non-carrier). CI = confidence interval. SE = standard error.

**Estrogen receptor status**	**β (95%CI)**	**SE**	**p**
Age at diagnosis (years)	0.01 (-0.04 – 0.07)	0.03	0.61
BRCA1	15.23 (-128 – inf)	$>10^3$	0.99
BRCA2	0.94 (-0.78 – 3.87)	1.07	0.38
**Progesterone receptor status**	**β (95%CI)**	**SE**	**p**
Age at diagnosis (years)	0.001 (-0.03 – 0.03)	0.02	0.93
BRCA1	-0.82 (-2.06 – 0.71)	0.68	0.23
BRCA2	-0.22 (-1.01 – 0.65)	0.42	0.60
**Human epidermal growth factor receptor 2**	**β (95%CI)**	**SE**	**p**
Age at diagnosis (years)	-0.004 ()	0.02	0.77
BRCA1	0.24	0.79	0.76
BRCA2	0.36	0.39	0.35
